# Supplementary material for: Increased glutarate production by blocking the glutaryl-CoA dehydrogenation pathway and a catabolic pathway involving l-2-hydroxyglutarate
Source: Nat Commun. 2018 May 29;9:2114. doi: 10.1038/s41467-018-04513-0 (PMC5974017; doi:10.1038/s41467-018-04513-0)
Supplement: Supplementary file 1 — Supplementary Information [file 41467_2018_4513_MOESM1_ESM.doc]

**Increased glutarate production by blocking the glutaryl-CoA dehydrogenation pathway and a catabolic pathway involving l-2-hydroxyglutarate**

Zhang *et al.*

**Supplementary Methods**

**Materials**

Glutarate, 2-ketoglutarate (2-KG), succinate, l-2-hydroxyglutarate disodium salt (l-2-HG), d-2-hydroxyglutarate disodium salt (d-2-HG), 5-aminovalerate, 2-aminoadipate, l-lysine, glutaryl-coenzyme A, adipate, l-malate, d-malate, l-lactate, d-lactate, l-2-hydroxybutyrate, 3-(4,5-dimethylthiazol-2-yl)-2,5-diphenyltetrazolium bromide (MTT), nitro blue tetrazolium (NBT), dichlorophenol-indophenol (DCPIP), coenzyme Q_10_, cytochrome *c*, resazurin, and diaphorase were purchased from Sigma-Aldrich (USA). d,l-2-Hydroxyglutarate disodium salt (2,3,3-D3) was purchased from Cambridge Isotope Laboratories, Inc. (USA). Yeast extract powder and tryptone were purchased from Oxoid Limited (United Kingdom). All other chemicals were of analytical reagent grade.

**Enzymatic assay of HGDH**

The gene encoding NAD^+^-dependent d-2-hydroxyglutarate dehydrogenase (HGDH) from *A. fermentans* was synthesized by General Biosystems, Inc. (Anhui, China) and ligated into expression plasmid pETDuet-1. The expression and purification procedures of HGDH were the same as CsiD and LhgO of *P. putida* KT2440. The activity of HGDH was measured in 100 μL solution containing 75 μL assay solution and 25 μL sample. The assay solution contained 100 mM HEPES (pH 8.0), 100 μM NAD^+^, 0.1 μg HGDH, 5 μM resazurin and 0.01 U mL^-1^ diaphorase (Sigma-Aldrich, USA). After 75 μL assay solution was added to 25 μL sample and incubated in the dark for 30 min in black 96-well plates (PerkinElmer, USA), fluorescence was measured using a fluorescence microplate reader (PerkinElmer, USA) with excitation at 540 ± 10 nm and emission of 610 ± 10 nm.

**Construction of standard curves for qPCR**

The partial *csiD*, *gdh* and *lhgO* gene sequences were amplified by PCR using the appropriate primers *csiD*-RTF/*csiD*-RTR, *gdh*-RTF/*gdh*-RTR, and *lhgO*-RTF/*lhgO*-RTR (**Supplementary Data 2**). The PCR products were cloned into a pEASY-Blunt Simple Cloning Vector (Tranrgen, China), generating Blunt-*csiD*, Blunt-*gdh* and Blunt-*lhgO*, respectively. The plasmids were extracted using a Plasmid Miniprep Kit (Biomiga, USA) and quantified by NanoDrop ND-1000 (Thermo Scientific, USA). The copy number of the plasmids were calculated based on the molecular weight of the recombinant plasmids^1^. A 10-fold serial dilution series of the recombinant plasmids, ranging from 1 × 10^3^ to 1 × 10^8^ copies μL^-1^, was used to construct the standard curves for *csiD*, *gdh* and *lhgO*. The standard curves were drawn by plotting the natural log of the threshold cycle (C_T_) against the natural log of the copy numbers of molecules using OriginPro software 8.0 (OriginLab, USA) (**Supplementary Fig. 13**). From the slope of each standard curve, PCR amplification efficiency (*E*) was calculated according to the equation: *E* = 10^−1/slope^ – 1^2^. All of the reactions were run in triplicate. Controls with no template were included for each reaction on the same plate.

**Sample preparation for qPCR**

*P. putida* KT2440 was grown overnight in LB medium, collected by centrifugation, washed with normal saline and then inoculated into MSM with 5 g L^-1^ glucose as the sole carbon source. Cells of *P. putida* KT2440 in MSM with 5 g L^-1^ glucose as the sole carbon source were harvested at the exponential growth phase, washed three times and inoculated into MSM with 5 g L^-1^ glutarate as the sole carbon source. Cells of *P. putida* KT2440 were immediately harvested and termed as the sample at 0 h. Samples were collected at timed intervals for the quantification of mRNA. Experiments were performed using three independent biological replicates.

**Carbon starvation experiments**

*P. putida* KT2440 was grown overnight in 5 mL LB medium at 200 rpm and 30 °C. The overnight cultures were harvested by centrifugation, washed twice and diluted to OD_600_ = 0.07 into 50 mL fresh glucose medium (MSM with 4 g L^-1^ glucose as the sole carbon source). When the culture was growing to OD_600_ = 1, the cells were harvested by centrifugation at 6,000 × *g* for 10 min, washed three times, then resuspended in 50 mL carbon starvation medium (MSM without carbon source) or glucose medium. Samples were harvested for qPCR assays of *csiD* and *gdh*. Experiments were performed using three independent biological replicates.

**Modeling of bacterial growth**

Modeling of bacterial growth were conducted using Gompertz equation as modified by Zwietering *et al*[^3^](#_ENREF_7). The lag time (λ), growth rate during exponential growth period (R), and maximal specific growth rates (μ_max_) were derived from the fitted model for each strain.

**Complementation of *csiD* and *gdh* in Δ*gdh*Δ*csiD***

To construct pMMB66EH-Gm, we amplified the gentamicin-resistant gene through PCR with primer pairs Gm-F (PvuI)/Gm-R (HindIII) using pUC18-mini-Tn*7*-Gm as a template, and inserted into the PvuI and HindIII sites of pMMB66EH. For the complementation of *csiD* and *gdh* in *P. putida* KT2440 (Δ*gdh*Δ*csiD*), fragments containing *csiD* and *gdh* were amplified by PCR using the *P. putida* KT2440 genome as the template with primer pairs *csiD*-cF/*csiD*-cR and *gdh*-cF/*gdh*-cR, respectively. The PCR products were cloned into the vectors pMMPc-Gm[^4^](#_ENREF_8) (containing the constitutive promoter *P_c_*) and pMMB66EH-Gm (containing the inducible promoter *P_tac_*) to contruct recombinant plasmids pMMPc-Gm*-csiD*, pMMPc-Gm-*gdh*, pMMPtac-Gm*-csiD*, and pMMPtac-Gm*-gdh*. The recombinant plasmids were then introduced into *P. putida* KT2440 (Δ*gdh*Δ*csiD*) by electroporation[^4^](#_ENREF_8).

**Quantification of l-lysine and l-2-HG**

The concentration of l-lysine was quantified using a HPLC system (Agilent 1100 series, Agilent Technologies, USA) equipped with a ZORBAX SB-C18 (5 μm, 4.6 mm × 150 mm) and a UV-Vis detector as described previously^5^. To extract intracellular l-2-HG, three separate cell cultures were sampled (15 mL) and centrifugated at 6,000 × *g* for 10 min at −9 °C. The pellets were washed with pre-chilled PBS (5 mL) and immediately frozen in liquid nitrogen. Samples were then extracted with 1 mL ethanol at 90 °C for 15 min[^6^](#_ENREF_9). The supernatants were obtained by centrifugation at 12,000 × *g* and 4 °C for 5 min and completely dried in a vacuum centrifuge (60 °C). The dried residue was resuspended in distilled water and stored at −80 °C until analysis. We assumed that the cell volume of a 1 mL *Pseudomonas* sample at an optical density (600 nm) of 1 was 3.5 μL[^7^](#_ENREF_10). Then, the intracellular l-2-HG concentration was estimated by dividing the amount of l-2-HG in the sample by the total cell volume. To measure extracellular l-2-HG, the supernatants of 1 mL cluture after centrifugation were stored at −20 °C for further analysis.

l-2-HG was quantified using liquid chromatography tandem mass spectrometry (LC-MS/MS) system. The LC-MS/MS analysis was carried out on an API 4000 tandem triple-quadrupole mass spectrometer (AB SCIEX, USA) in negative ion mode and coupled with an Agilent 1200 series liquid chromatograph (Agilent Technologies, USA). The mobile phase used for the enantiomeric separation for 2-HG consisted of a mixture of: (A) 0.1% triethylamine adjusted to pH 4.5 with acetic acid; and (B) methanol (95:5). The column used was a Chirobiotic R column of 250 mm length × 4.6 mm I.D., 5 mm silica gel particles bonded to the macrocyclic glycopeptide ristocetin A (Supelco Analytical, USA). The flow rate was 0.5 mL min^-1^ with no split. The total run time was 12 min. d,l-2-Hydroxyglutarate disodium salt (2,3,3-D3) was used as internal standard (ISTD). Monitored transitions were 147.0 → 129.0 for l-2-HG and 150.0 → 132.0 for ISTD. The concentrations of l-2-HG is calculated using a standard curve which is assayed with each run.

**Determination of intracellular NAD**^+^ **and NADH concentrations**

Three separate cell cultures of *P. putida* KT2440 with different rotational speeds were sampled (1 mL) and centrifugated at 13,000 × *g* for 1 min. The pellets were resuspended in 300 μl 0.2 M sodium hydroxide (NaOH) for NADH extraction or 0.2 M hydrochloric acid (HCl) for NAD^+^ extraction, incubated at 50 °C for 10 min, then subjected to further extraction and determination using NAD^+^/NADH Quantification Kit (Biovision, USA) according to the reagent specification.


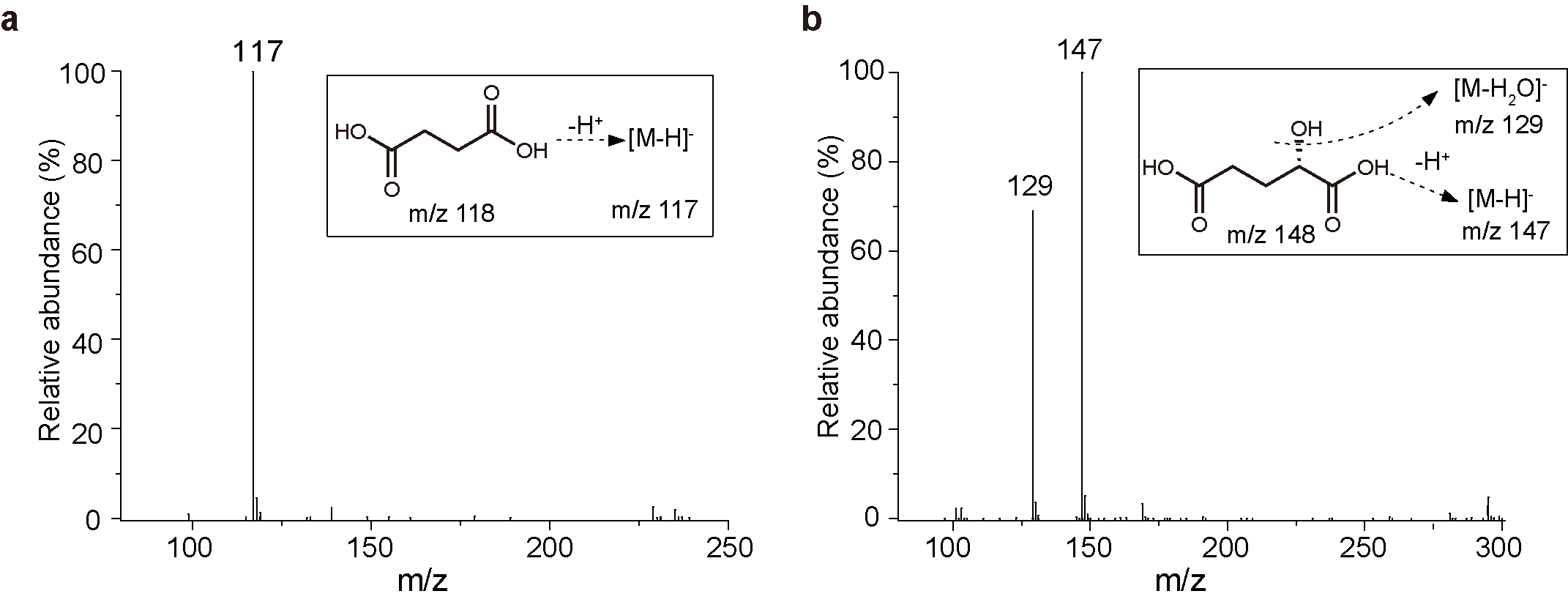


**Supplementary Figure 1. Mass spectra analysis of the products catalyzed by CsiD.** (**a**) Mass spectra of the production of succinate catalyzed by CsiD. The proposed fragmentation patterns of succinate are shown in the upper right. (**b**) Mass spectra of production of 2-hydroxyglutarate catalyzed by CsiD. The proposed fragmentation patterns of 2-hydroxyglutarate are shown in the upper right.


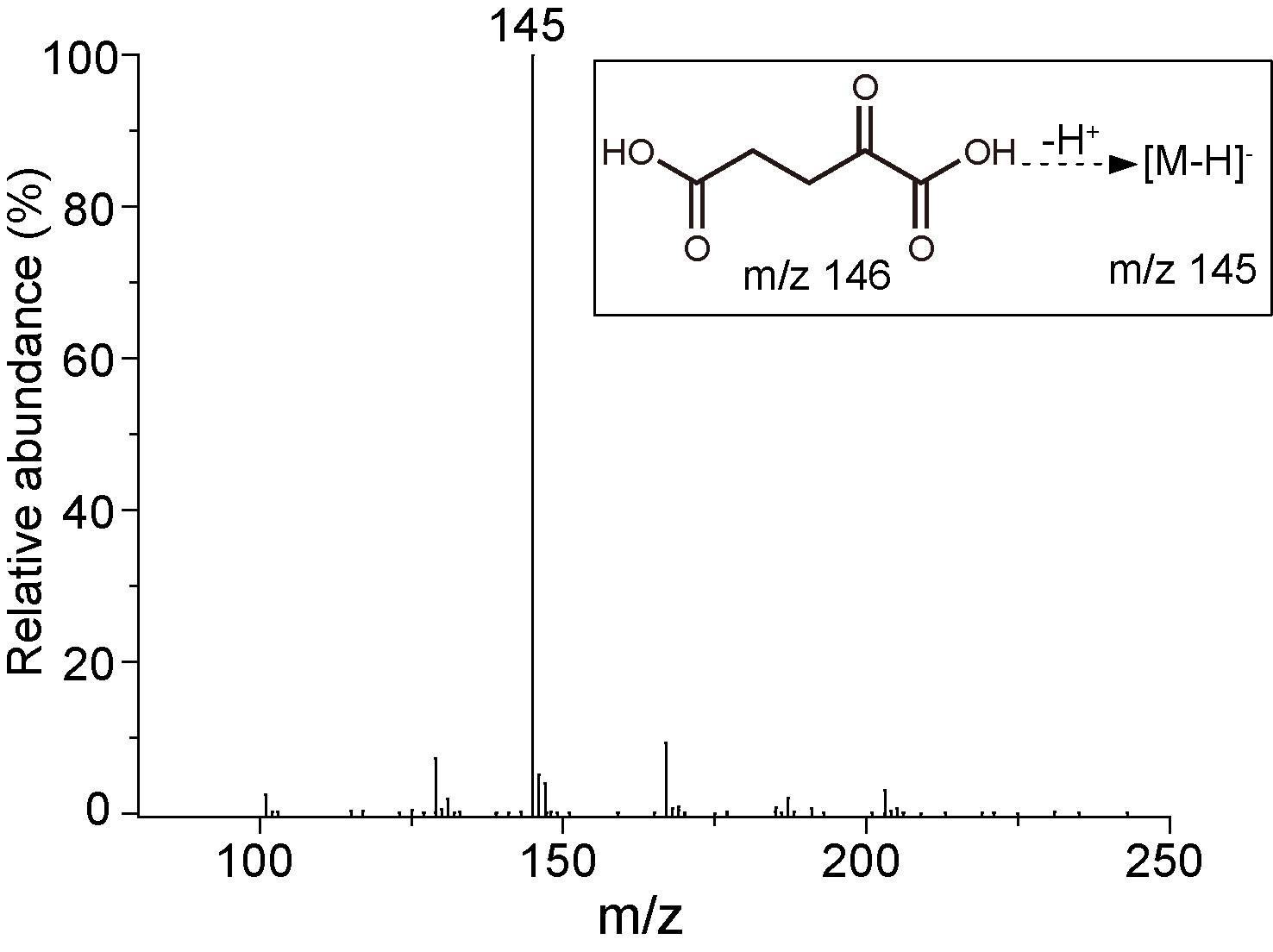


**Supplementary Figure 2. Mass spectra of the production of 2-KG catalyzed by LhgO.** The proposed fragmentation patterns of 2-KG are shown in the upper right.


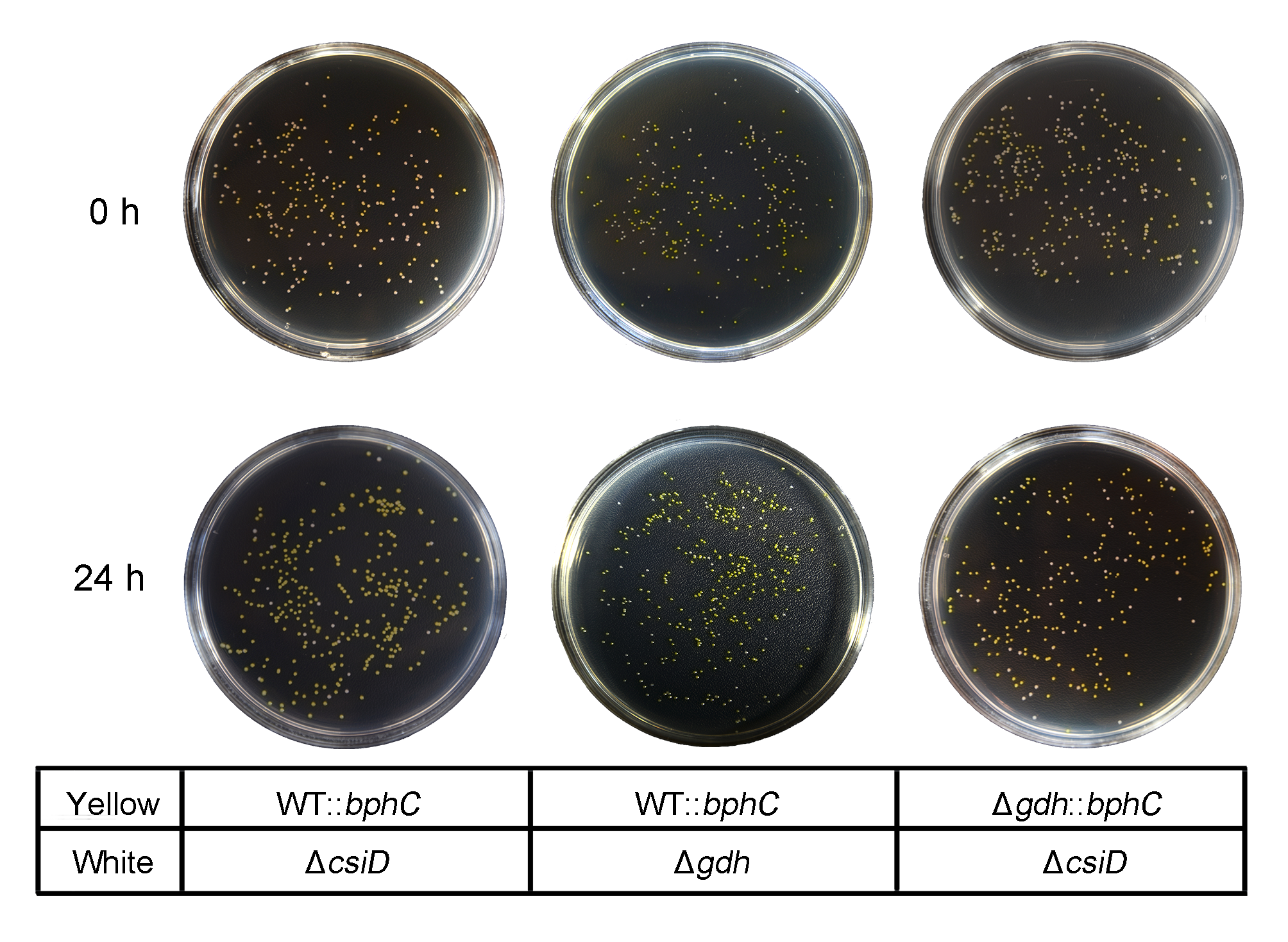


**Supplementary Figure 3. The color reactions of bacterial colonies in competitive fitness assays at 0 h and 24 h.** The competitors marked with *bphC* gene colored yellow to catechol and the unmarked competitors were not.


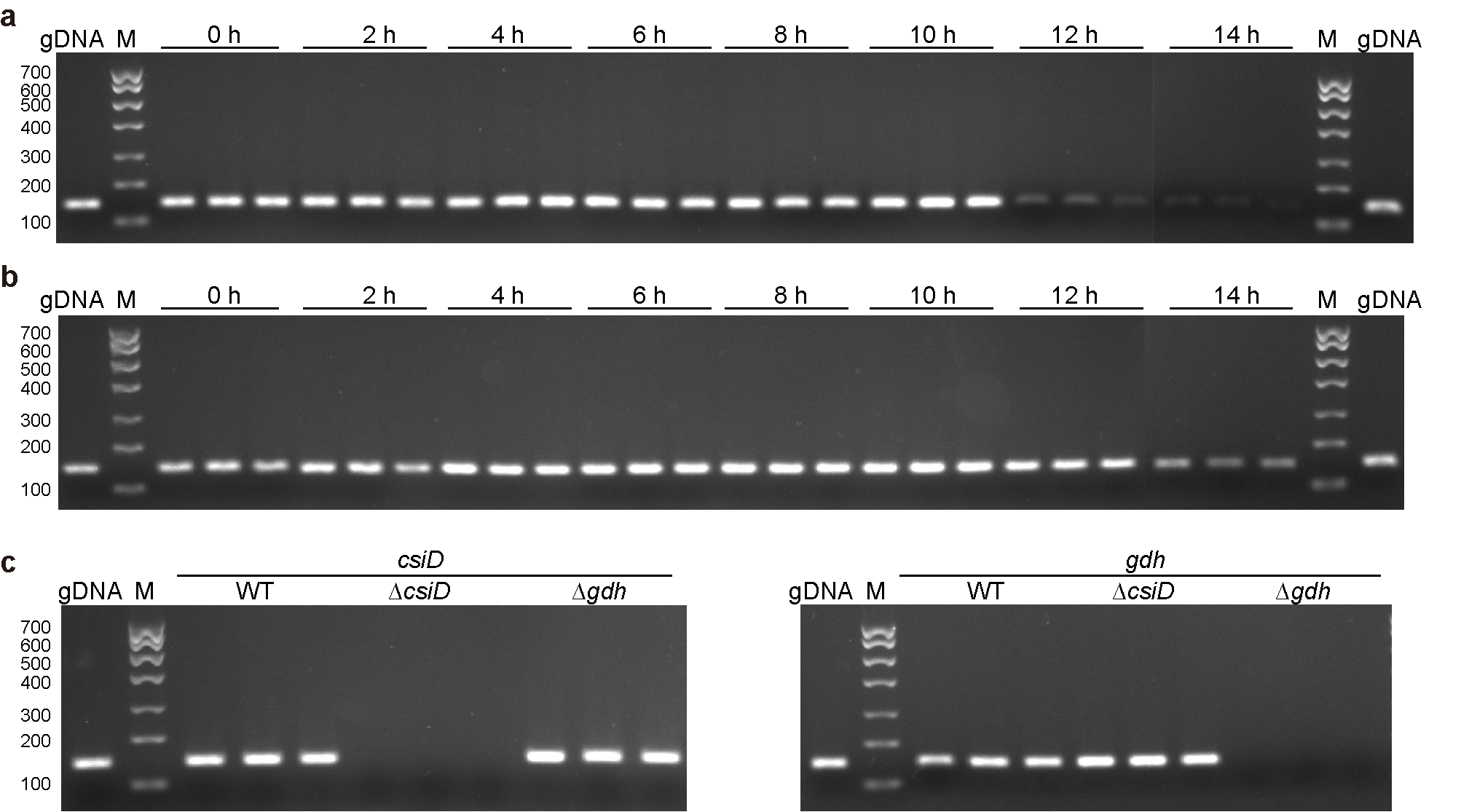


**Supplementary Figure 4. Agarose gel electrophoresis of *csiD* and *gdh* RT-PCR products of *P. putida* KT2440 during the growth in glutarate.** (**a**) *csiD* RT-PCR products of *P. putida* KT2440 during the growth in glutarate at different time points. (**b**) *gdh* RT-PCR products of *P. putida* KT2440 during the growth in glutarate at different time points. (**c**) *csiD* and *gdh* RT-PCR products of *P. putida* KT2440, *P. putida* KT2440 (Δ*csiD*) and *P. putida* KT2440 (Δ*gdh*) during the growth in glutarate at 0 h. Genomic DNA of *P. putida* KT2440 (gDNA) was used as a positive control. There are three independent biological replicates in the experiments. Numbers on the left present the sizes of the markers (in base pairs).


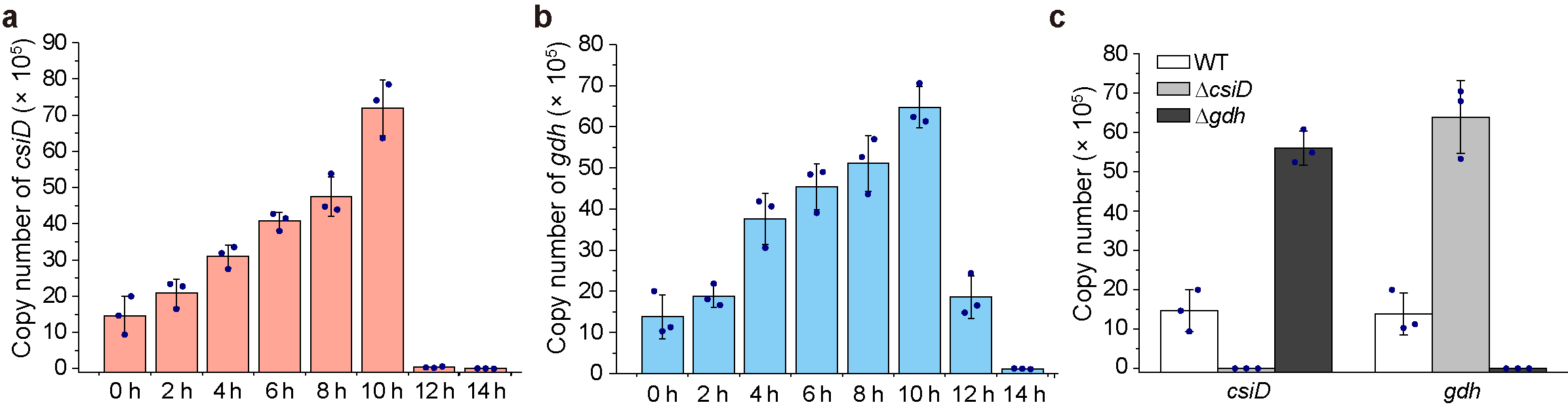


**Supplementary Figure 5. Expression of *csiD* and *gdh* in *P. putida* KT2440 during the growth in glutarate**. (**a**) The copy number of *csiD* of *P.* *putida* KT2440 during the growth in glutarate. (**b**) The copy number of *gdh* of *P. putida* KT2440 during the growth in glutarate. (**c**) The copy numbers of *csiD* and *gdh* of *P. putida* KT2440, *P. putida* KT2440 (Δ*csiD*) and *P. putida* KT2440 (Δ*gdh*) at the beginning of growth (0 h) in glutarate. Data shown are mean ± s.d. (*n* = 3 independent experiments).


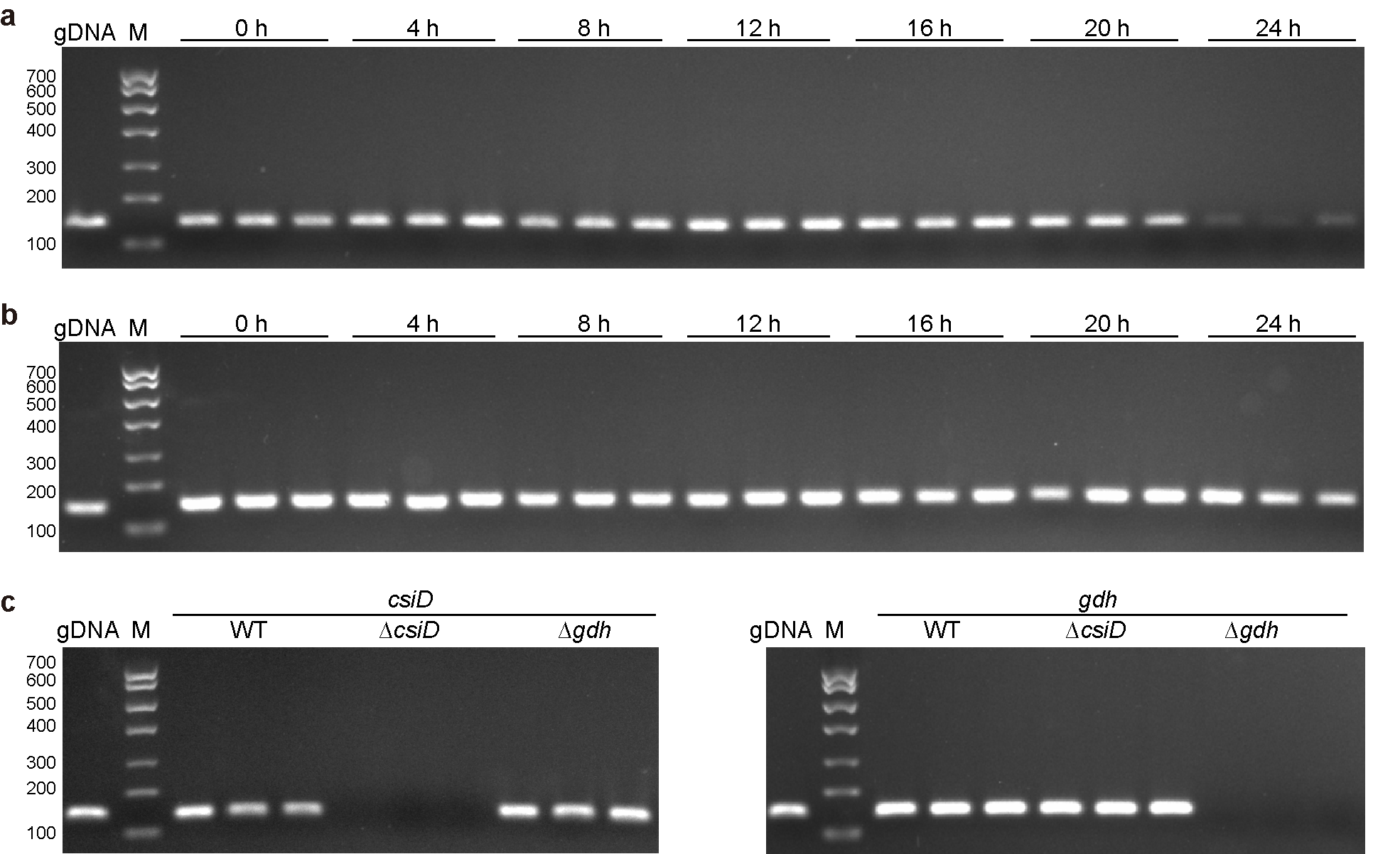


**Supplementary Figure 6. Agarose gel electrophoresis of *csiD* and *gdh* RT-PCR products of *P. putida* KT2440 during the growth in lysine.** (**a**) *csiD* RT-PCR products of *P. putida* KT2440 during the growth in lysine at different time points. (**b**) *gdh* RT-PCR products of *P. putida* KT2440 during the growth in lysine at different time points. (**c**) *csiD* and *gdh* RT-PCR products of *P. putida* KT2440, *P. putida* KT2440 (Δ*csiD*) and *P. putida* KT2440 (Δ*gdh*) during the growth in lysine at 0 h. Genomic DNA of *P. putida* KT2440 (gDNA) was used as a positive control. There are three independent biological replicates in the experiments. Numbers on the left present the sizes of the markers (in base pairs).


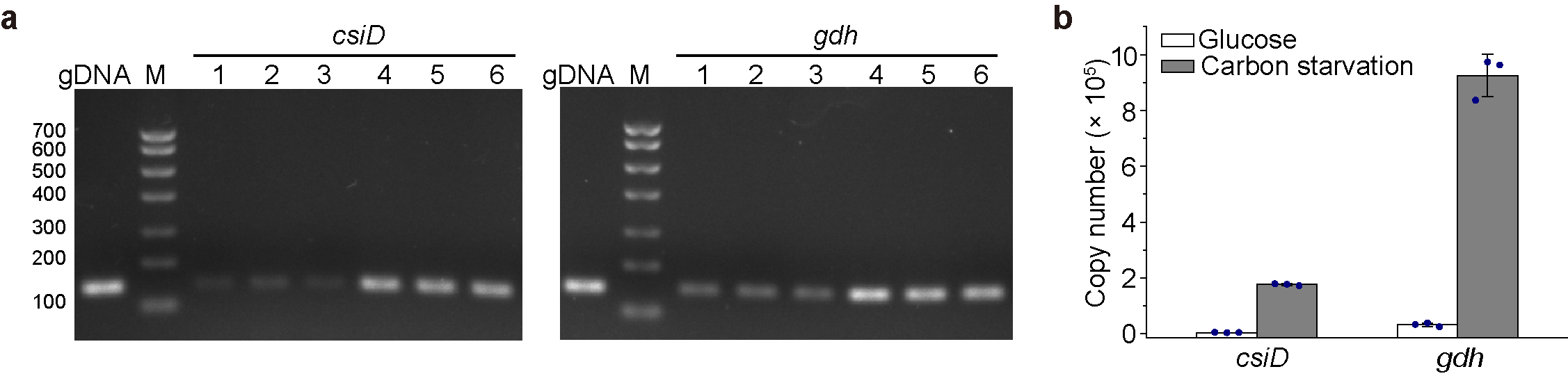


**Supplementary Figure 7. Expression of *csiD* and *gdh* genes in *P. putida* KT2440 under carbon starvation.** (**a**) The *csiD* and *gdh* RT-PCR products of *P. putida* KT2440 during the growth in glucose medium (lanes 1−3) and carbon starvation medium (lanes 4−6). Genomic DNA of *P. putida* KT2440 (gDNA) was used as a positive control. There are three independent biological replicates in the experiments. Numbers on the left present the sizes of the markers (in base pairs). (**b**) The copy numbers of *csiD* and *gdh* of *P. putida* KT2440 during the growth in glucose medium and carbon starvation medium at 0 h. Data shown are mean ± s.d. (*n* = 3 independent experiments).


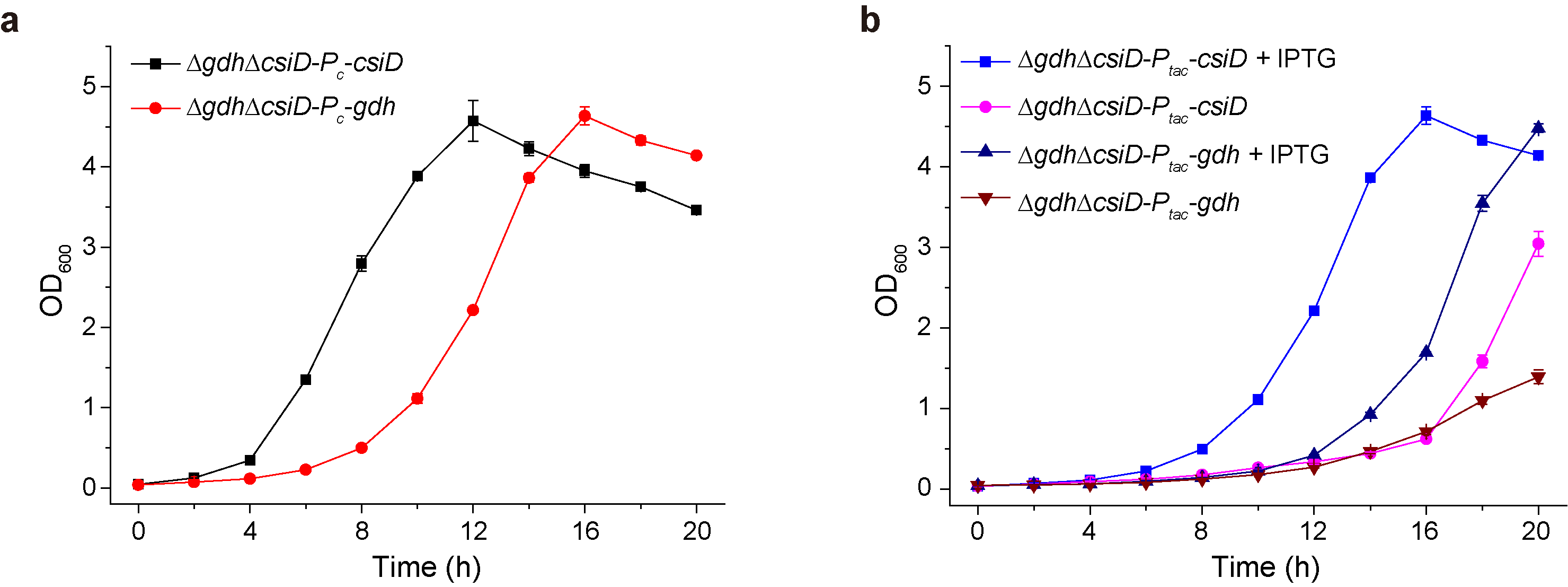


**Supplementary Figure 8. Growth curves of *csiD* and *gdh* complementary strains of *P. putida* KT2440 (Δ*gdh*Δ*csiD*) in medium with glutarate as the sole carbon source.** The expression of *csiD* and *gdh* is under the regulation of the constitutive promoter *P_c_* (**a**) or the inducible promoter *P_tac_* (**b**). IPTG was added (shown as +IPTG) or not when the inducible promoter *P_tac_* were used in the experiments. Data shown are mean ± s.d. (*n* = 3 independent experiments).


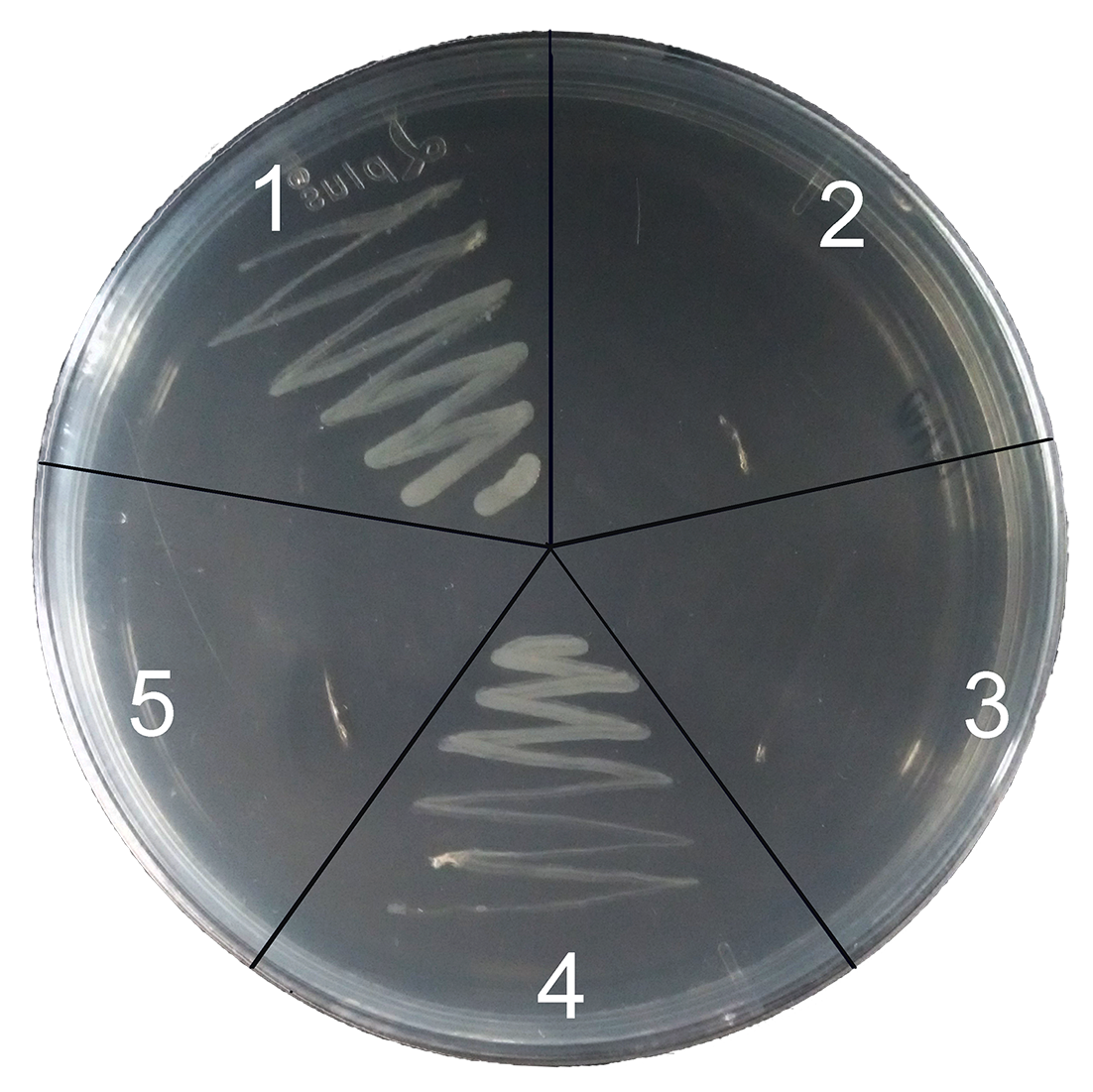


**Supplementary Figure 9. Growth of *P. putida* KT2440 and its derivatives.** Solid MSM containing 5 g L^-1^ glutarate was the sole carbon source. Picture was taken after 12 h. 1. *P. putida* KT2440; 2. *P. putida* KT2440 (Δ*csiD*); 3. *P. putida* KT2440 (Δ*csiD*Δ*lhgO*); 4. *P. putida* KT2440 (Δ*gdh*); 5. *P. putida* KT2440 (Δ*gdh*Δ*csiD*).


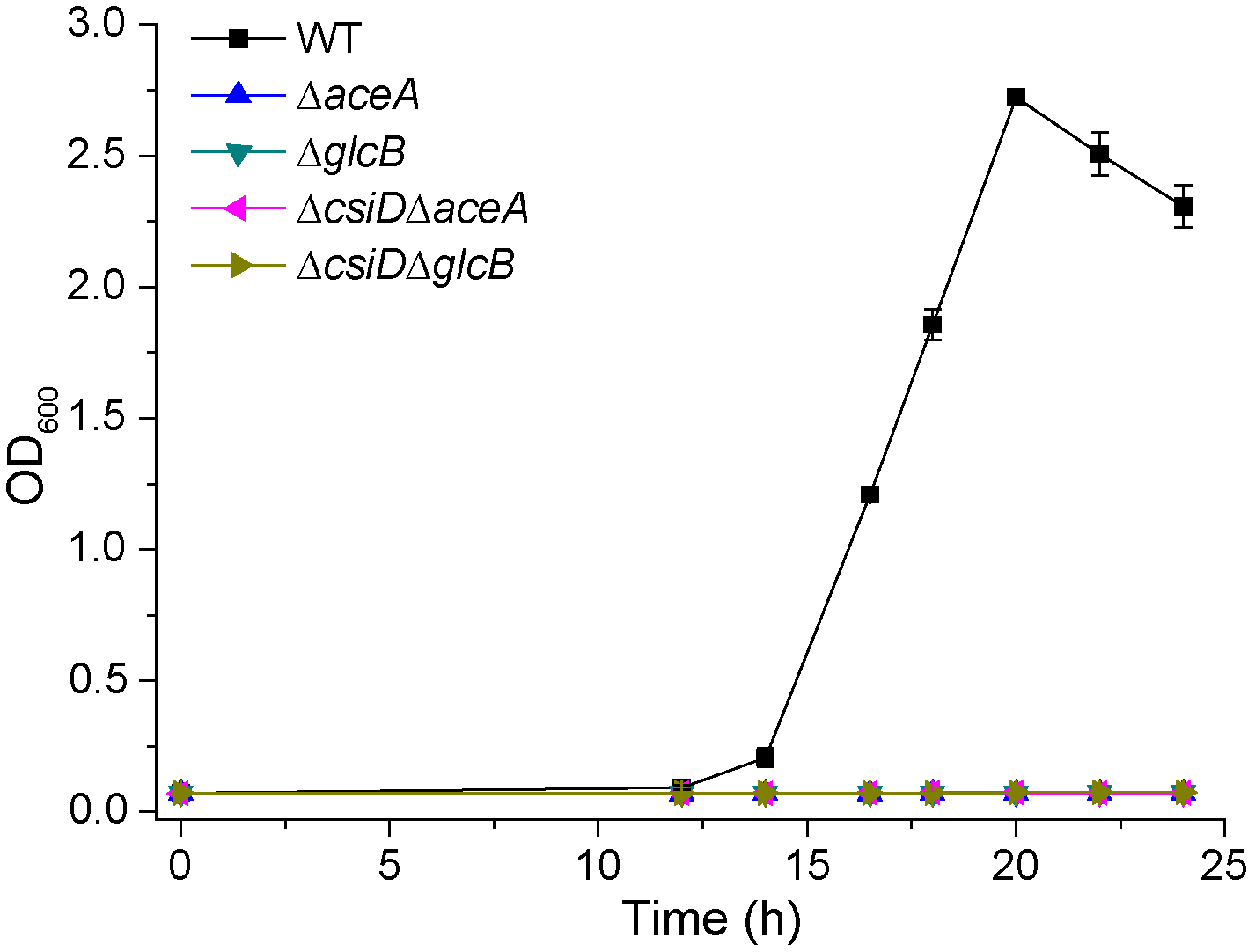


**Supplementary Figure 10. Growth of *P. putida* KT2440 and its glyoxylate cycle deletion mutants.** MSM containing 5 g L^-1^ acetate was the sole carbon source. Data shown are mean ± s.d. (*n* = 3 independent experiments).


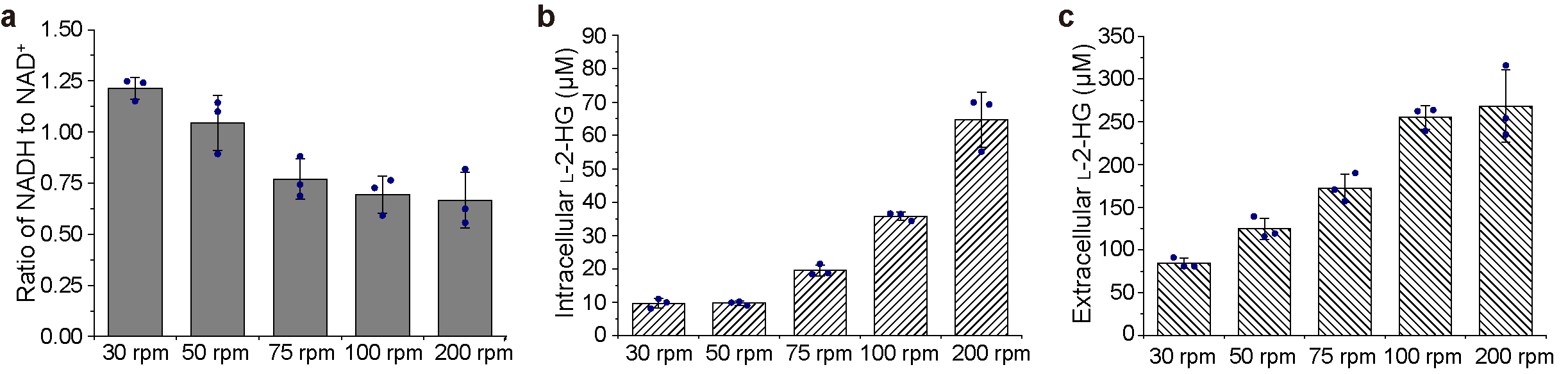


**Supplementary Figure 11. The intracellular redox state and l-2-HG generation in *P. putida* KT2440 at different rotational speeds.** (**a**) The ratio of NADH to NAD^+^ in *P .putida* KT2440 at different rotational speeds. (**b**) The concentrations of intracellular l-2-HG at different rotational speeds. (**c**) The concentrations of extracellular l-2-HG at different rotational speeds. Data shown are mean ± s.d. (*n* = 3 independent experiments).


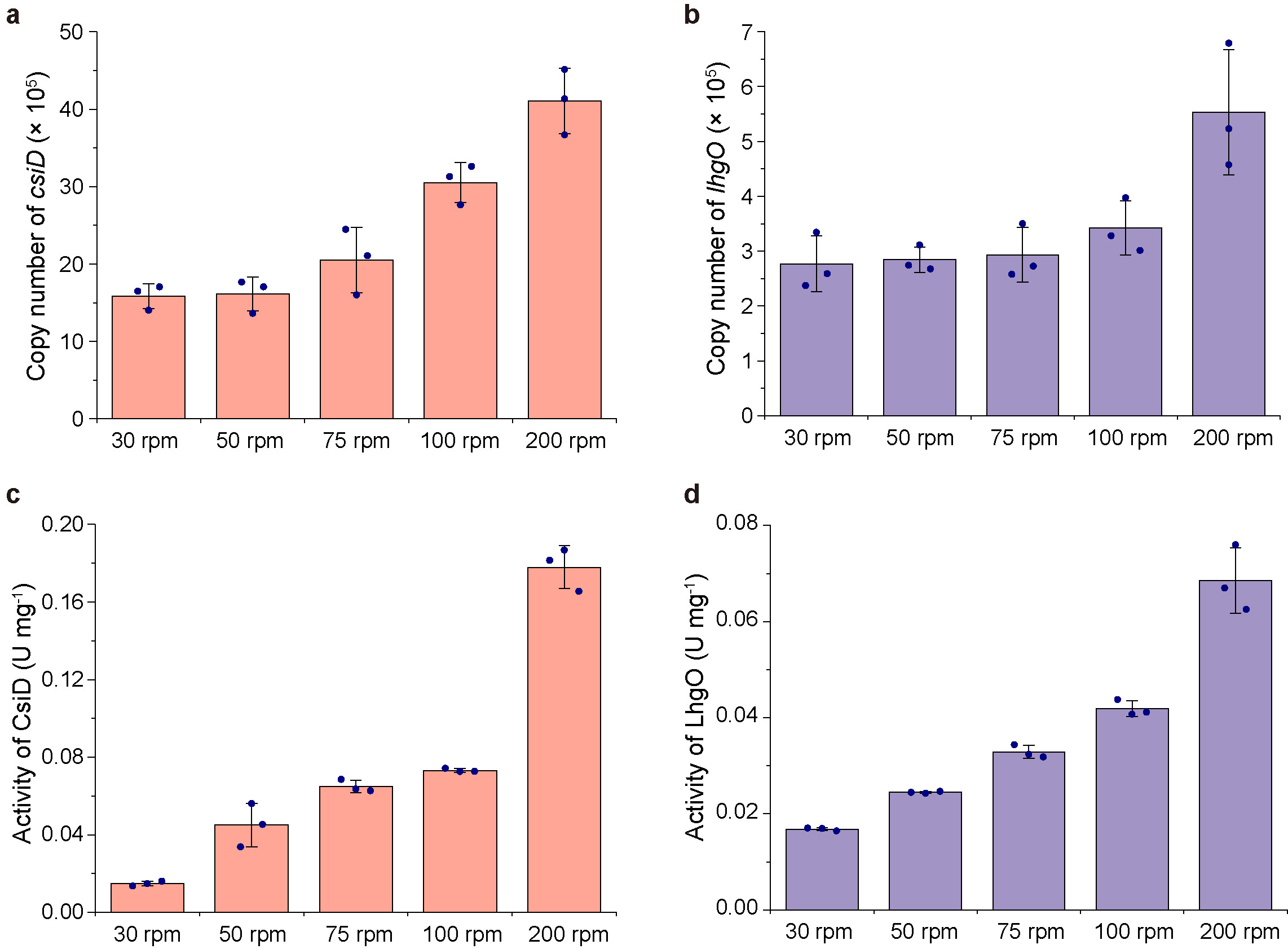


**Supplementary Figure 12. The expression and activity of CsiD and LhgO in *P. putida* KT2440 at different rotational speeds.** The copy numbers of *csiD* (**a**) and *lhgO* (**b**) at different rotational speeds in glutarate medium were analyzed by qPCR. (**c**) The activity of CsiD at different rotational speeds. (**d**) The activity of LhgO at different rotational speeds. Data shown are mean ± s.d. (*n* = 3 independent experiments).


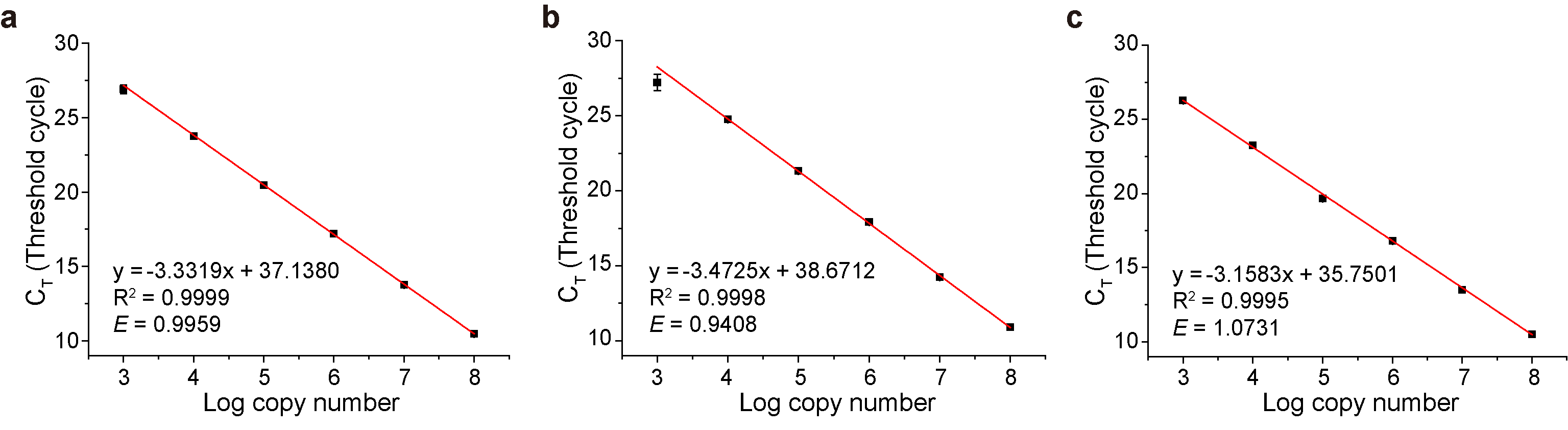


**Supplementary Figure 13. Construction of the standard curves using absolute quantitative real-time PCR. (a) *csiD*, (b) *gdh*, and (c) *lhgO***. The standard curves were constructed with serial 10-fold dilutions of each recombinant plasmid, ranging from 1 × 10^3^ to 1 × 10^8^ copies μL^-1^. For each set, determined C_T_ values were plotted against the logarithm of their known initial copy number (per μL). The best-fit equations and amplification efficiency (*E*) are also shown.

**Supplementary Table 1. Growth test of *P. putida* strains in glutarate medium^a^**

| Strain | λ (h) | R (h^-1^) | μ_max_ (h^-1^) | Mb (OD_600_) | Vs (g L^-1^ h^-1^) |
| --- | --- | --- | --- | --- | --- |
| Wild-type | 2.17 ± 0.07 | 0.63 ± 0.02 | 0.76 ± 0.02 | 4.51 ± 0.08 | 0.43 ± 0.02 |
| KT2440 (∆*gdh*) | 2.10 ± 0.09 | 0.59 ± 0.02 | 0.71 ± 0.02 | 3.96 ± 0.07 | 0.40 ± 0.00 |
| KT2440 (∆*csiD*) | 5.00 ± 0.06 | 0.28 ± 0.00 | 0.34 ± 0.01 | 4.06 ± 0.12 | 0.26 ± 0.01 |

^a^λ, lag time; R, growth rate during exponential growth period; μ_max_, the maximum specific growth rate; Mb, the obtained maximum biomass; Vs, the average velocity of substance consumption. Data shown are mean ± s.d. (*n* = 3 independent experiments).

**Supplementary Table 2. Occurrence of genes enconding CsiD, LhgO and GDH in bacterial genomes^a^**

| **Organism** | **CsiD** | **LhgO** | **GDH** |
| --- | --- | --- | --- |
| **Gammaproteobacteria** | **453** | **538** | **332** |
| *Pseudomonas putida* KT2440 | **+** | **+** | **+** |
| *Pseudomonas aeruginosa* PAO1 | **−** | **−** | **+** |
| *Halomonas campaniensis* LS21 | **+** | **+** | **+** |
| *Escherichia coli* K-12 | **+** | **+** | **−** |
| *Salmonella enterica* | **+** | **+** | **−** |
| *Klebsiella pneumoniae* | **+** | **+** | **−** |
| **Alphaproteobacteria** | **0** | **65** | **226** |
| *Vibrio cholerae* | **−** | **+** | **−** |
| *Brucella abortus* | **−** | **+** | **+** |
| **Betaproteobacteria** | **0** | **4** | **309** |
| *Paraburkholderia phytofirmans* PsJN | **−** | **+** | **+** |
| *Burkholderia pseudomallei* K42 | **−** | **−** | **+** |
| **Deltaproteobacteria** | **0** | **0** | **9** |
| **Epsilonproteobacteria** | **1** | **1** | **0** |
| *Helicobacter felis* ATCC 49179 | **+** | **+** | **−** |
| **Actinobacteria** | **0** | **4** | **14** |
| *Nocardia cyriacigeorgica* GUH-2 | **−** | **+** | **−** |
| *Amycolatopsis mediterranei* U32 | **−** | **−** | **+** |
| **Bacteroidetes** | **0** | **0** | **100** |
| **Acidobacteria** | **0** | **0** | **3** |
| **Gemmatimonadetes** | **0** | **0** | **2** |
| **Gemmatimonadetes** | **0** | **0** | **7** |
| **Ignavibacteriae** | **0** | **0** | **2** |
| **Total** | **454** | **612** | **1004** |

^a^Representative species in several taxonomic groups of bacteria are shown as rows and the presence or absence of genes enconding the respective functional protein (columns) is shown by **+** or **−**. Numbers for taxonomic group rows indicate the number of species that have a gene ortholog.

**Supplementary Table 3.** **Specific activities of CsiD and LhgO in different strains**

|  | Specific activity (U mg^-1^)^a^ |  |
| --- | --- | --- |
| Strain | CsiD | LhgO |
| *P. putida* KT2440 | 176.77 ± 2.56 | 1.64 ± 0.01 |
| *E. coli* K-12 MG1655 | 126.82 ± 0.40 | 1.54 ± 0.11 |
| *K. pneumoniae* ATCC25955 | 118.08 ± 0.36 | 1.69 ± 0.02 |
| *S. typhimurium* CT18 | 194.24 ± 0.26 | 2.43 ± 0.14 |

^a^ Data shown are mean ± s.d. (*n* = 3 independent experiments).

**Supplementary Table 4. List of strains and corresponding protein sequences information**

|  | **Accession number** |  |  |
| --- | --- | --- | --- |
| **Organism** | **CsiD** | **LhgO** | **GDH** |
| *Pseudomonas putida* KT2440 | AAN68517.1 | AAN68518.1 | AAN65791.1 |
| *Pseudomonas plecoglossicida* NyZ12 | AJG13158.1 | AJG13159.1 | AJG16758.1 |
| *Pseudomonas aeruginosa* W60856 | ALY86977.1 | ALY86976.1 | ALY86759.1 |
| *Pseudomonas citronellolis* P3B5 | AMO77848.1 | AMO77847.1 | AMO79185.1 |
| *Pseudomonas fluorescens* UK4 | AIG01260.1 | AIG01261.1 | AIG04057.1 |
| *Pseudomonas knackmussii* B13 | CDF85356.1 | CDF85355.1 | CDF86684.1 |
| *Pseudomonas monteilii* SB3078 | AHC83078.1 | AHC83077.1 | AHC85446.1 |
| *Pseudomonas monteilii* SB3101 | AHC88454.1 | AHC88453.1 | AHC90814.1 |
| *Pseudomonas mosselii* SJ10 | AIN58563.1 | AIN58562.1 | AIN61369.1 |
| *Pseudomonas putida* DLL-E4 | AHZ77777.1 | AHZ77776.1 | AHZ74664.1 |
| *Pseudomonas putida* BIRD-1 | ADR60439.1 | ADR60438.1 | ADR57896.1 |
| *Pseudomonas putida* DOT-T1E | AFO46332.1 | AFO46331.1 | AFO46437.1 |
| *Pseudomonas putida* F1 | ABQ78914.1 | ABQ78913.1 | ABQ76352.1 |
| *Pseudomonas putida* GB-1 | ABY98768.1 | ABY98767.1 | ABY96091.1 |
| *Pseudomonas putida* H8234 | AGN81961.1 | AGN78939.1 | AGN77264.1 |
| *Pseudomonas putida* HB3267 | AGA73880.1 | AGA73879.1 | AGA71144.1 |
| *Pseudomonas putida* NBRC 14164 | BAN54763.1 | BAN54762.1 | BAN52005.1 |
| *Pseudomonas putida* ND6 | AFK72222.1 | AFK72223.1 | AFK69630.1 |
| *Pseudomonas putida* S12 | AJA16778.1 | AJA16198.1 | AJA13384.1 |
| *Pseudomonas putida* S13.1.2 | AJQ51172.1 | AJQ46805.1 | AJQ49671.1 |
| *Pseudomonas putida* S16 | AEJ13551.1 | AEJ13550.1 | AEJ10714.1 |
| *Pseudomonas* *resinovorans* NBRC 106553 | BAN50702.1 | BAN50701.1 | BAN45848.1 |
| *Pseudomonas* sp. FGI182 | AHD14425.1 | AHD14426.1 | AHD12286.1 |
| *Pseudomonas* sp. TKP | AHC35734.1 | AHC35733.1 | AHC32726.1 |
| *Pseudomonas* sp. VLB120 | AGZ37289.1 | AGZ37288.1 | AGZ32990.1 |
| *Halomonas campaniensis* LS21 | AIA76435.1 | AIA76436.1 | AIA75089.1 |

**Supplementary Table 5.** List of protein sequences information included in the phylogenetic analyses of CsiD

| **Organism** | **GenBank accession number** |
| --- | --- |
| *Pseudomonas putida* KT2440 | AAN68517.1 |
| *Pseudomonas plecoglossicida* | AJG13158.1 |
| *Pseudomonas aeruginosa* | ALY86977.1 |
| *Pseudomonas citronellolis* | AMO77848.1 |
| *Pseudomonas fluorescens* | AIG01260.1 |
| *Pseudomonas knackmussii* | CDF85356.1 |
| *Pseudomonas monteilii* | AHC88454.1 |
| *Pseudomonas mosselii* | AIN58563.1 |
| *Pseudomonas* *resinovorans* | BAN50702.1 |
| *Halomonas campaniensis* | AIA76435.1 |
| *Escherichia coli* | AML00809.1 |
| *Klebsiella pneumoniae* | AEW59682.1 |
| *Salmonella enterica* | CAD05898.1 |
| *Klebsiella variicola* | ADC60006.1 |
| *Citrobacter freundii* | AHY10017.1 |
| *Obesumbacterium proteus* | AMO80787.1 |
| *Hafnia alvei* | AIU74202.1 |
| *Enterobacter aerogenes* | CCG31546.1 |
| *Salmonella bongori* | AGR59995.1 |
| *Citrobacter koseri* | ABV15078.1 |
| *Shigella boydii* | ABB67379.1 |
| *Enterobacter lignolyticus* | ADO47146.1 |
| *Leclercia adecarboxylata* | ALZ98127.1 |
| *Escherichia albertii* | AHE61188.1 |
| *Shigella flexneri* | AMM78297.1 |
| *Shimwellia blattae* | AFJ48128.1 |
| *Chromohalobacter salexigens* | ABE59347.1 |

**Supplementary Table 6. List of protein sequences information included in the phylogenetic analyses of Lhg**O

| **Organism** | **GenBank accession number** |
| --- | --- |
| *Pseudomonas putida* KT2440 | AAN68518.1 |
| *Pseudomonas plecoglossicida* | AJG13159.1 |
| *Pseudomonas aeruginosa* | ALY86976.1 |
| *Pseudomonas citronellolis* | AMO77847.1 |
| *Pseudomonas fluorescens* | AIG01261.1 |
| *Pseudomonas knackmussii* | CDF85355.1 |
| *Pseudomonas monteilii* | AHC88453.1 |
| *Pseudomonas mosselii* | AIN58562.1 |
| *Pseudomonas* *resinovorans* | BAN50701.1 |
| *Pseudomonas entomophila* | CAK14582.1 |
| *Pseudomonas chlororaphis* | AKA24768.1 |
| *Pseudomonas denitrificans* | AGI24943.1 |
| *Pseudomonas parafulva* | AIZ32300.1 |
| *Pseudomonas alkylphenolia* | AIL60795.1 |
| *Pseudomonas trivialis* | AKS04829.1 |
| *Pseudomonas protegens* | AGL84263.1 |
| *Halomonas campaniensis* | AIA76436.1 |
| *Vibrio cholerae* | ABQ19305.1 |
| *Brucella abortus* | AKO30023.1 |
| *Escherichia coli* | AML00808.1 |
| *Klebsiella pneumoniae* | AEW59681.1 |
| *Salmonella enterica* | CAD05899.1 |
| *Homo sapiens* | NP_079160.1 |
| *Pan troglodytes* | XP_001154304.2 |
| *Macaca mulatta* | XP_001099959.1 |
| *Canis lupus familiaris* | XP_863530.2 |
| *Bos taurus* | NP_001094560.1 |
| *Mus musculus* | NP_663418.1 |
| *Rattus norvegicus* | NP_001101498.1 |
| *Gallus gallus* | XP_421462.2 |
| *Danio rerio* | NP_001139067.1 |
| *Drosophila melanogaster* | NP_609923.2 |
| *Anopheles gambiae* | XP_317624.2 |
| *Caenorhabditis elegans* | NP_503730.1 |
| *Xenopus tropicalis* | XP_002938171.1 |
| *Arabidopsis thaliana* | NP_191243.1 |
| *Kluyveromyces lactis* | XP_454615.1 |
| *Magnaporthe oryzae* | NP_191243.1 |
| *Neurospora crassa* | XP_962718.2 |
| *Oryza sativa Japonica Group* | NP_001044579.1 |

**Supplementary References**

1. Whelan, J.A., Russell, N.B. & Whelan, M.A. A method for the absolute quantification of cDNA using real-time PCR. *J. Immunol. Methods* **278**, 261−269 (2003).

2. Lee, C., Kim, J., Shin, S.G. & Hwang, S. Absolute and relative QPCR quantification of plasmid copy number in *Escherichia coli*. *J. Biotechnol*. **123**, 273−280 (2006).

3. Zwietering, M.H., Jongenburger, I., Rombouts, F.M. & Van, t.R.K. Modeling of the bacterial growth curve. *Appl. Environ. Microbiol.* **56**, 1875 (1990).

4. Xu, Y., Tao, F., Ma, C. & Xu, P. New constitutive vectors: useful genetic engineering tools for biocatalysis. *Appl. Environ. Microbiol.* **79**, 2836−2840 (2013).

5. Liu, P. *et al.* Enzymatic production of 5-aminovalerate from l-lysine using l-lysine monooxygenase and 5-aminovaleramide amidohydrolase. *Sci. Rep.* **4**, 5657 (2014).

6. Winder, C.L. *et al.* Global metabolic profiling of *Escherichia coli* cultures: an evaluation of methods for quenching and extraction of intracellular metabolites. *Anal. Chem.* **80**, 2939−2948 (2008).

7. Volkmer, B. & Heinemann, M. Condition-dependent cell volume and concentration of *Escherichia coli* to facilitate data conversion for systems biology modeling. *PLoS One* **6**, e23126 (2011).
