# Supplementary material for: Increased glutarate production by blocking the glutaryl-CoA dehydrogenation pathway and a catabolic pathway involving l-2-hydroxyglutarate
Source: Nat Commun. 2018 May 29;9:2114. doi: 10.1038/s41467-018-04513-0 (PMC5974017; doi:10.1038/s41467-018-04513-0)
Supplement: Supplementary file 2 — Description of Additional Supplementary Files [file 41467_2018_4513_MOESM2_ESM.doc]

**Description of Additional Supplementary Files**

File Name: Supplementary Data 1

Description: Strains and plasmids used in this study

File Name: Supplementary Data 2

Description: Oligonucleotides used in this study
